# Supplementary material for: Daily Nutritional Supplementation with Vitamin D3 and Phenylbutyrate to Treatment-Naïve HIV Patients Tested in a Randomized Placebo-Controlled Trial
Source: Nutrients. 2019 Jan 10;11(1):133. doi: 10.3390/nu11010133 (PMC6356462; doi:10.3390/nu11010133)
Supplement: Supplementary file 1 [file nutrients-11-00133-s001.pdf]

Online Supplementary Materials

1. Methods

1.1. Partnership

This study was conducted in collaboration between Addis Ababa University in Addis Ababa, Ethiopia, and the Karolinska Institutet in Stockholm, Sweden. Study subjects were recruited from the Tikur Anbessa University Hospital (Black Lion Specialized Hospital), while the laboratory work of the study was performed in collaboration with the Armauer Hansen Research Institute (AHRI) and the International Clinical Laboratory (ICL) in Addis Ababa. ICL is a Randox International Quality Assessment Scheme (RIQAS)-accredited and Centers for Disease Control and Prevention (CDC)-certified commercial laboratory in Addis Ababa, Ethiopia.

1.2. Ethical approvals

The clinical trial was conducted in agreement with the principles of the Declaration of Helsinki and the Guidelines for Good Clinical Practice. Ethical approval in Ethiopia was obtained both at national and regional levels (from the University hospital and research laboratory) including the National Research Ethics Review Committee (NRERC; 3.10/608/04) and the Institutional Review Boards (IRB; 008/11/IM) at Addis Ababa University and at the Armauer Hansen Research Institute (AHRI; P031/11) and also from the Food, Medicine and Health Care Administration and Control Authority of Ethiopia (Medical Product Agency, FMHACA; U2/6/22/12). Ethical approval for analyses of clinical trial samples in Sweden was obtained from the local ethical review board (EPN; 2011/1014-31/1), in Stockholm.

1.3. Interventions

The dosing scheme of vitD$_3$ and PBA was selected as follows: the dose of vitD$_3$ was based on a previous study from P. Bergman *et al.*, which showed that daily doses of vitD$_3$ (4000 IU/day) are required to enhance serum concentrations of 25(OH)D$_3$ to a level that is relevant to prevent respiratory tract infections [1]. The dose of PBA was chosen from a study conducted by M. Akhirunnesa *et al*, where 500 mg PBA given twice daily (together with 5000 IU vitD$_3$) to healthy volunteers proved to be the optimal oral dose to induce LL-37 in monocyte-derived macrophages obtained from PBA-treated individuals [2].

1.4. Randomization

Placebo tablets for vitD$_3$ and PBA had an identical appearance and taste as the corresponding active drug. Pharmacists at the Tikur Anbessa University Hospital prepared the study medication in polyethylene (PE)-bottles (Scandinavian Formulas) that were labeled with printed stickers (Merck) including the numbers from the randomization list. The pharmacists were the only staff members with access to the randomization list: treatment allocation was concealed from patients, primary investigators, and other staff. Coded, sealed, opaque envelopes containing the key to the patient IDs were stored separately from the randomization list.

1.5. HIV testing

HIV testing was made according to the protocol in the national guideline, using HIV (1+2) Antibody Colloidal Gold (KHB, Shanghai Kehua Bio-engineering Co Ltd, China) as a screening test, followed by HIV 1/2 STAT-PAK (Chembo Diagnostics, USA) if KHB was positive. If the result of STAT-PAK was discordant with KHB, a third test, Unigold HIV (Trinity Biotech, Ireland), was used as a tie breaker to determine the result. The correlation between HIV viral load and CD4$^+$ T cell counts in HIV-positive patients was determined at baseline (Supplementary figure 1).

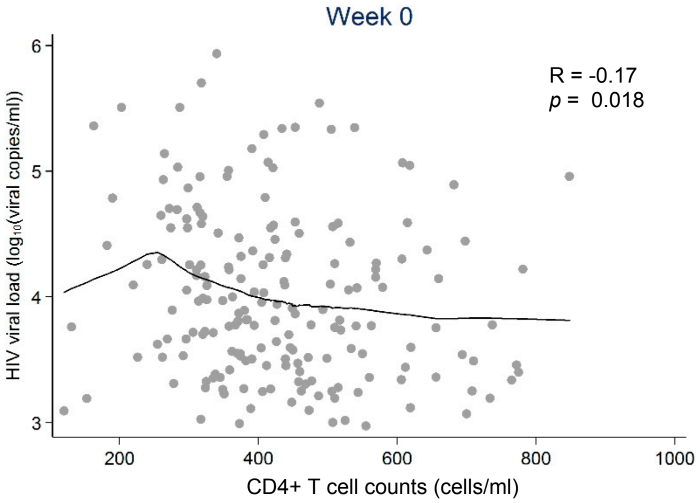

Supplementary figure 1. Correlation analysis. Inverse correlation between HIV viral load and $CD4^+$ T cell counts in HIV-positive study subjects at baseline.

1.6. Adverse events (AE)

Solicited and unsolicited AEs were monitored during the complete study period including clinical examinations to record HIV-specific clinical complications and laboratory tests to record liver function: serum alanine aminotransferase (ALT), aspartate aminotransferase (AST), total bilirubin and alkaline phosphatase (ALP); kidney function: serum creatinine and urea; and calcium/phosphate homeostasis: serum calcium, phosphate, and albumin. Blood chemistry analyses were conducted using kits from BD (ESR, bilirubin) and Abbott Diagnostics (IL, USA). AEs were graded according to DAIDS AE grading Table (The Division of AIDS Table for Grading the Severity of Adult and Pediatric Adverse Events) [3]. Both clinical and laboratory AEs were graded as mild (grade 1), moderate (grade 2), severe (grade 3) and potentially life-threatening (grade 4). The physiological state of each patient was monitored by the laboratory analysis shown in Table S1. The clinical and laboratory safety data was reviewed by an external data and safety monitoring board (DSMB) appointed at the Tikur Anbessa University Hospital in Addis Ababa, Ethiopia. None of the AEs had any connection to the study drugs (vitD₃ or PBA), as reviewed by the DSMB.

Supplementary Table 1. Blood chemistry data

**Variables**[1]	**Week**	**Placebo (n = 102)**	**VitD₃+PBA (n = 95)**	***p*-value**
CD4 T cell counts	0	412 (340, 505)	409 (320, 517)	
(cells/ml)	4	409 (312, 502)	396 (325, 538)	0.83
	8	392 (324, 486)	379 (284, 499)	0.46
	16	377 (315, 467)	374 (280, 530)	1.00
	24	383 (294, 528)	371 (281, 484)	0.54
WBC	0	5.2 (4.1, 6.3)	5.5 (4.3, 6.7)	
(SI units)	4	5.2 (4.6, 6.3)	5.3 (4.3, 6.8)	0.73
	8	5.4 (4.3, 6.5)	5.1 (4.1, 6.3)	0.59
	16	5.0 (4.0, 6.2)	5.0 (4.4, 6.3)	0.54
	24	5.2 (4.3, 6.5)	5.4 (4.3, 6.1)	0.66
ESR	0	30 (18, 42)	34 (20, 48)	
(mm/hour)	4	28 (18, 40)	38 (20, 50)	0.03
	8	33 (18, 48)	36 (22, 50)	0.11
	16	32 (20, 44)	32 (20, 44)	0.90
	24	31 (20, 43)	38 (21, 48)	0.36
Hemoglobin	0	13.7 (12.4, 14.6)	13.6 (12.4, 14.6)	
(mg/dl)	4	13.8 (12.9, 14.8)	13.6 (12.8, 15.0)	0.46
	8	13.4 (12.7, 14.6)	13.4 (12.5, 14.2)	0.39
	16	13.7 (12.5, 14.5)	13.6 (13.0, 14.3)	0.98
	24	13.8 (12.8, 14.8)	13.9 (12.9, 14.8)	0.96
Albumin	0	4.0 (3.7, 4.2)	4.0 (3.6, 4.2)	
(g/l)	4	3.9 (3.7, 4.1)	3.9 (3.7, 4.1)	0.89

	8	3.9 (3.7, 4.1)	3.8 (3.6, 4.0)	0.23
	16	3.8 (3.6, 4.1)	3.8 (3.6, 4.1)	0.63
	24	3.8 (3.6, 4.0)	3.8 (3.6, 4.1)	0.46
Calcium	0	9.0 (8.4, 9.5)	8.8 (8.3, 9.3)	
(g/dl)	4	9.0 (8.5, 9.4)	9.1 (8.6, 9.5)	0.90
	8	8.9 (8.4, 9.3)	8.9 (8.3, 9.3)	0.96
	16	8.7 (8.4, 9.4)	9,0 (8.4, 9.6)	0.18
	24	8.7 (8.2, 9.4)	9.0 (8.4, 9.9)	0.02
Phosphate	0	3.4 (3.2, 3.8)	3.6 (3.0, 3.9)	
(mg/dl)	4	3.3 (2.9, 3.7)	3.5 (3.1, 3.8)	0.007
	8	3.4 (3.1, 3.8)	3.6 (3.2, 3.9)	0.05
	16	3.3 (3.0, 3.6)	3.4 (3.1, 3.8)	0.06
	24	3.5 (3.2, 3.8)	3.4 (3.1, 3.8)	0.59
Creatinine	0	0.7 (0.6, 0.8)	0.7 (0.6, 0.8)	
(mg/dl)	4	0.7 (0.6, 0.8)	0.7 (0.6, 0.8)	0.57
	8	0.7 (0.6, 0.8)	0.7 (0.6, 0.8)	0.84
	16	0.7 (0.6, 0.8)	0.7 (0.6, 0.9)	0.65
	24	0.7 (0.6, 0.8)	0.8 (0.7, 0.9)	0.09
Bilirubin (total)	0	0.51 (0.4, 0.7)	0.50 (0.4, 0.6)	
(mg/dl)	4	0.50 (0.4, 0.7)	0.50 (0.4, 0.6)	0.58
	8	0.50 (0.4, 0.6)	0.43 (0.4, 0.6)	0.43
	16	0.50 (0.4, 0.6)	0.46 (0.3, 0.6)	0.75
	24	0.48 (0.4, 0.6)	0.47 (0.4, 0.6)	0.99
Urea	0	18 (14, 21)	18 (15, 23)	
(mg/dl)	4	16 (14, 22)	17 (14, 22)	0.88
	8	16 (14, 20)	18 (14, 22)	0.31
	16	17 (14, 22)	18 (14, 23)	0.51
	24	18 (13, 22)	17 (14, 21)	0.48
ALT	0	20 (15, 27)	18 (15, 25)	
(IU/l)	4	18 (15, 24)	18 (15, 24)	0.90
	8	18 (15, 24)	18 (15, 23)	0.66
	16	18 (14, 25)	16 (15, 22)	0.44
	24	17 (14, 22)	17 (14, 22)	0.69
AST	0	24 (20, 31)	23 (19, 29)	
(IU/l)	4	23 (18, 29)	22 (18, 30)	0.83
	8	24 (20, 27)	22 (18, 30)	0.44
	16	21 (18, 28)	21 (18, 26)	0.76
	24	21 (18, 27)	22 (17, 27)	0.50
ALP	0	72 (58, 89)	71 (57, 92)	
(mg/dl)	4	71 (55, 86)	69 (57, 85)	0.97
	8	68 (53, 88)	64 (53, 83)	0.53
	16	67 (55, 85)	67 (56, 77)	0.67
	24	68 (53, 81)	66 (54, 81)	0.72

[1] Data are median (25th, 75th percentile).
WBC, white blood cell; ESR, erythrocyte sedimentation rate; ALT, alanine aminotransferase;
AST, aspartate aminotransferase; ALP, alkaline phosphatase

1.7. Statistical analysis

Following the intention-to-treat (ITT)-concept, subjects who discontinued treatment but provided follow-up values were analyzed as though they were still part of the treatment group. In this study, we used a modified ITT (mITT) analysis, generally defined as a subset of the ITT population that allows exclusion of some randomized subjects in a justified way. Modified ITT is commonly used when the disease diagnosis or results used to determine inclusion/exclusion criteria are not immediately available at randomization. Therefore, reasons for patients to be excluded from ITT could be that tests after randomization show that the patient is misdiagnosed and/or ineligible. mITT is commonly used in antimicrobial/anti-infective trials, when test results are often obtained after randomization. Accordingly, while rapid HIV tests are available, determination of $CD4^+$ T cell counts and viral load may take time, especially in a resource-poor setting. The randomization and the start of treatment cannot wait until the results used to determine the different inclusion and exclusion criteria are available. In the mITT analyses, multiple imputation [4], as opposed to single imputation or last observation carried forward, ensures that standard errors and *P*-values are not artificially deflated [5].

Supplemental References

1. Bergman, P.; Norlin, A.C.; Hansen, S.; Rekha, R.S.; Agerberth, B.; Bjorkhem-Bergman, L.; Ekstrom, L.; Lindh, J.D.; Andersson, J. Vitamin D3 supplementation in patients with frequent respiratory tract infections: a randomised and double-blind intervention study. *BMJ Open* **2012**, *2*, doi:10.1136/bmjopen-2012-001663e001663 [pii]bmjopen-2012-001663 [pii].
2. Mily, A.; Rekha, R.S.; Kamal, S.M.; Akhtar, E.; Sarker, P.; Rahim, Z.; Gudmundsson, G.H.; Agerberth, B.; Raqib, R. Oral intake of phenylbutyrate with or without vitamin D3 upregulates the cathelicidin LL-37 in human macrophages: a dose finding study for treatment of tuberculosis. *BMC Pulm Med* **2013**, *13*, 23, doi:10.1186/1471-2466-13-231471-2466-13-23 [pii].
3. National Institute of Allergy and Infectious Diseases (NIAID). D*ivision of AIDS Table for Grading the Severity of Adult and Pediatric Adverse Events*. December, 2004.
4. Sterne JA, White IR, Carlin JB et al. Multiple imputation for missing data in epidemiological and clinical research: potential and pitfalls. *BMJ* 2009;338:b2393.
5. Turnbull BW. Nonparametric estimation of a survivorship function with doubly censored data. *J Am Stat Assoc* 1974;69:169-73.
